# Myocardial bridging causing ischemia and recurrent chest pain: a case report

**DOI:** 10.1186/1755-7682-4-24

**Published:** 2011-07-07

**Authors:** Mohamed Abdou

**Affiliations:** 1 Cardiology Department, Zagazig Faculty of Medicine, Zagazig, Egypt

## Abstract

**Background:**

Myocardial bridging is present when a segment of a major epicardial coronary artery runs intramurally through the myocardium. It usually has a benign prognosis, but in some cases myocardial ischemia, infarction and sudden cardiac death have been reported. We are here reporting a case of myocardial bridging which was complicated with recurrent chest pain and transient ST-segment elevation during exercise treadmill test.

**Case presentation:**

A 40 year-old-man presented with recurrent retrosternal chest pain of 2 months duration. He had history of smoking and was obese, otherwise no physical abnormalities were detected by examination. Electrocardiogram and blood tests were normal apart from impaired glucose tolerance with elevated triglycerides and decreased level of high density lipoprotein cholesterol. While doing exercise treadmill test, the patient developed chest pain and significant ST-segment elevation in almost all precordial leads that persisted for about 15 minutes through recovery. We decided to admit the patient to the coronary care unit for further management and to perform coronary angiogram. Myocardial bridging was observed in the mid segment of the left anterior descending coronary artery. Medical treatment was decided.

At one year follow up, our patient was healthy and had no cardiac complaints. In conclusion, myocardial bridging may predispose to coronary vasospasm that may leads to ischemic complications.

## Background

The major coronary arteries are located in the sub-epicardial region [1]. Localization of a major coronary arterial segment, the 'tunnelled artery', in the myocardial tissue is termed myocardial bridging; a congenital coronary anomaly [2]. In these patients, there is a temporary systolic coronary arterial luminal narrowing. Symptomatic patients are most often middle-aged men with typical or atypical chest pain, either related or unrelated to exercise [1,3]. Myocardial bridging usually has a benign prognosis, but some cases associated with myocardial ischemia [4], infarction [5], coronary spasm [6], arrhythmias [7], and sudden death [8] have been reported.

## Case presentation

A 40 year-old-man was evaluated at our out patient clinic complaining of recurrent compressing retrosternal chest pain of 2 months duration. That pain occurred at rest or with exertion and sometimes awake the patient from sleep. He had history of smoking but no history of diabetes, hypertension, dyslipidemia, and no family history of coronary artery disease. His body mass index was 37 kg/m^2 ^and waist circumference 115 cm.

No physical abnormalities were detected by clinical examination and blood pressure was 130/85 mmHg.

Resting ECG was normal and blood tests revealed triglycerides 1.8 mmol/L, high density lipoprotein cholesterol 0.57 mmol/L, total cholesterol 4.15 mmol/L, low density lipoprotein cholesterol 2.77 mmol/L, fasting blood glucose 6.8 mmol/L, post prandial glucose 9.9 mmol/L, glycosylated hemoglobin (HbA1c) 7.0%. All other blood tests were normal.

**Figure 1:**
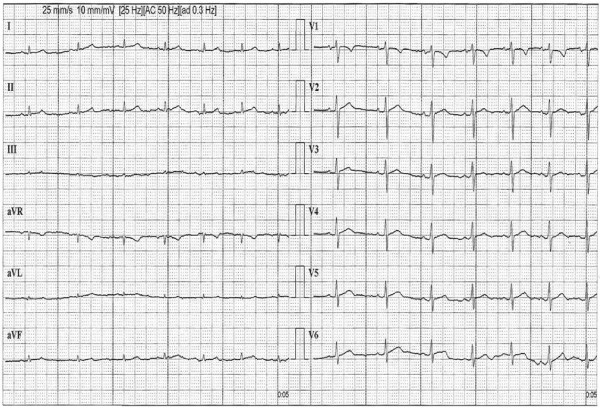
**Early phase of exercise treadmill test with normal tracing**.

**Figure 2:**
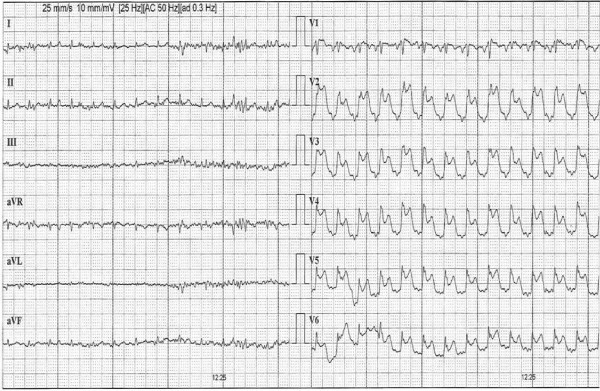
**Maximal exercise at which the test was terminated with ST-segment elevation in precordial leads**.

**Figure 3:**
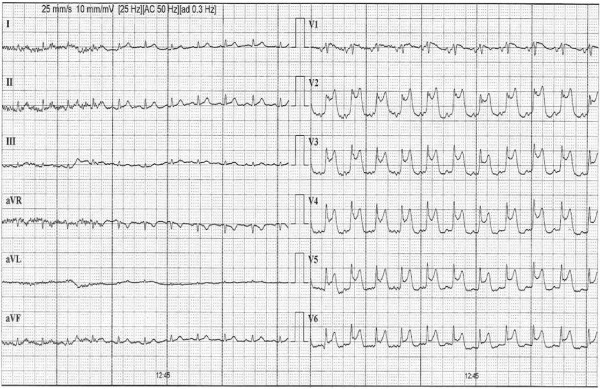
**Recovery phase of exercise treadmill test with still elevated ST-segment in precordial leads**.

**Figure 4:**
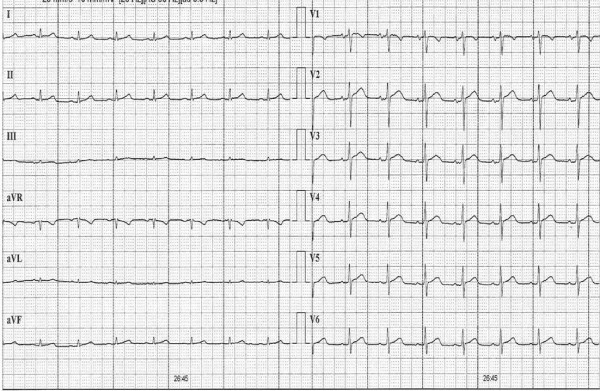
**Normalised ST-segment in precordial leads at late recovery**.

We decided to perform exercise stress test using CAEP protocol (The **C**hronotropic **A**ssessment **E**xercise **P**rotocol). Blood pressure, heart rate and 12-leads ECG were recorded at rest, at two-minute intervals during exercise, at peak exercise, and through the recovery phase. The ECG and ST-segment were continuously displayed and measured automatically by a computer-assisted system in all 12 leads. At heart rate of 144 beat/minute, the ST segment showed progressive elevation and the patient reported chest pain that necessitated termination of the test. The patient received oxygen and sublingual nitroglycerin and was reassured until disappearance of chest pain accompanied with complete recovery of ST-segment after about 15 minutes from cessation of exercise (Figures 1, 2, 3, 4).

The patient was transferred to the CCU for observation and medical treatment. He was given oral acetylsalicylic acid, 300 mg per day; clopidogril 300 mg 1^st ^day,75 mg thereafter; enoxaparin, 1 mg/kg/12 h; metoprolol, 50 mg per day;atorvastatin 20 mg per day. Serial cardiac enzymes values were normal.

**Figure 5:**
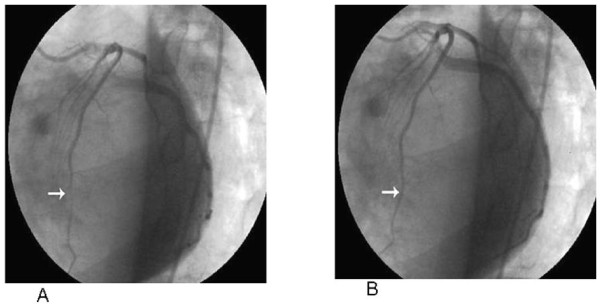
**Systolic compression at the mid-portion of LAD at the LAO view (A), with normalization of systolic compression (B)**.

Two days later, coronary angiography and left ventriculography were performed. The left coronary system was imaged at left and right oblique, right cranial and caudal and anteroposterior cranial positions. Significant coronary artery systolic luminal narrowing was observed in the mid segment of the left anterior descending coronary artery at left anterior oblique cranial position on coronary angiogram (Figure 5). The right coronary artery was normal and ventriculogram revealed preserved systolic function without wall motion abnormalities, or mitral regurge.

The patient was discharged on metoprolol 50 mg per day, oral acetylsalicylic acid, 81 mg per day, and atorvastatin 20 mg per day with complete instructions for risk factors modification.

At the one year follow up, our patient was healthy and had no cardiac complaints.

## Discussion

Myocardial bridging can be seen as an incidental finding at coronary arteriography. Previous studies have reported its prevalence at 1.5 to 16% when assessed by coronary angiography but in some autopsy series, it is as high as 80% [9,10]. Left anterior descending coronary artery was exclusively involved by 70% [11]. Because myocardial bridging is a common finding at autopsy of normal subjects, it had been thought to be a benign anatomic variation [12]. In spite that myocardial bridging rarely causes myocardial ischemia [11], previous reports have demonstrated its pathologic potential. Stable or unstable angina pectoris [4,13], acute myocardial infarction [5,14], complete atrioventricular block [7,15] or sudden death [8] associated with myocardial bridges have been described.

It is well known that the main pathogenesis of acute coronary syndrome consists of atherosclerotic plaque disruption and thrombus formation [16].

This was the provisional diagnosis in our patient as he was smoking and had criteria of cardiac metabolic syndrome. The second possibility was coronary spasm on atherosclerotic plaque. So, we recommended the CCU management for our patient. There is evidence that the arterial segment proximal to the myocardial bridge has a higher frequency of atherosclerosis, whereas the tunnelled segment is relatively spared despite evidence of endothelial dysfunction, which could predispose to coronary vasospasm and thrombosis [17,18].

The degree of coronary obstruction by the myocardial bridge depends on such factors as location superficial' or'deep', thickness, length of the muscle bridge, and degree of cardiac contractility [12]. Possible explanation of changes occurred during exercise in our patient could be severe coronary spasm depending on multiple of these factors. Vessel compression within the myocardial bridge is not solely a systolic event but persists throughout a large portion of diastole as evidenced with intravascular ultrasonography [19]. Although this malformation is present at birth, symptoms usually do not develop before the third decade, like the case in our report; the reason for this is not clear [12]. Coronary angiography was planned. However, no atherosclerotic plaque in the major coronary arteries was detected. There was temporary systolic coronary arterial luminal narrowing at the mid-portion of the left anterior descending coronary artery at left anterior oblique view. Therefore, we decided to follow the patient conservatively. At one year follow-up and without doing stress test, we obtained an excellent result at least in symptom relieve with risk factors modification, B-blokers, statins, and oral acetylsalicylic acid. Nitrates generally was avoided because they increase the angiographic degree of systolic narrowing and can lead to worsening of the symptoms [20]. On the other hand, ß-blockers decrease the tachycardia and increase diastolic time, with a decrease in contractility and compression of the coronary arteries. This was evidenced with intravenous injection of esmolol during tachypacing in symptomatic patients with severe myocardial bridging showing decrease Doppler flow velocities, with a return to baseline values and normalization of the diastolic-to-systolic flow velocity ratio within the bridged segment [21]. In spite that acetylsalicylic acid can worse coronary spasm, most in coronary arteries without atherosclerosis, even at low doses [22]. However, endothelial damage, platelet aggregation and thrombus formation may occur at the site of focal arterial constriction even when the reduction in transluminal diameter is insufficient to alter substantially the rate of flow [23] and hence acetylsalicylic acid can be useful specially in our patient with cardiac metabolic syndrome. Intracoronary stents, coronary artery bypass graft, and surgical myotomy may be the final way to treat but which patient should be selected for invasive or surgical therapy is still a matter of debate.

## Conclusion

Myocardial bridging may predispose to coronary vasospasm that may leads to ischemic complications. This report, together with those previously published, suggests that myocardial bridging may no longer be considered simply a benign variation of coronary anatomy. Inotropic challenge possibly should be considered for assessing the clinical importance of certain myocardial bridges. However, objective signs of ischemia cannot always be demonstrated in patients with myocardial bridging, most likely because of a large variability.

## Abbreviations

ECG: electrocardiogram; CCU: coronary care unit.

## Consent

Written informed consent was obtained from the patient for publication of this case report.

## Competing interests

The author declares that he has no competing interests.
